# Influence of the Gingival Condition on the Performance of Different Gingival Displacement Methods—A Randomized Clinical Study

**DOI:** 10.3390/jcm10132747

**Published:** 2021-06-22

**Authors:** Katharina Kuhn, Heike Rudolph, David Zügel, Benjamin A. Just, Michael Hrusa, Thomas Martin, Sigmar Schnutenhaus, Jens Dreyhaupt, Ralph G. Luthardt

**Affiliations:** 1 Department of Prosthetic Dentistry, Center of Dentistry, Ulm University, Albert-Einstein-Allee 11, 89081 Ulm, Germany; heike.rudolph@computerzaehne.de (H.R.); drzuegel@zahnarztpraxis-lenggries.de (D.Z.); bj@bestsmile.ch (B.A.J.); michael.hrusa@zahnaerzte-mutlangen.de (M.H.); info@zahnarzt-schwangau.de (T.M.); sigmar.schnutenhaus@uniklinik-ulm.de (S.S.); computerzaehne@computerzaehne.de (R.G.L.); 2 Institute of Epidemiology and Medical Biometry, Ulm University, Schwabstraße 13, 89075 Ulm, Germany; jens.dreyhaupt@uni-ulm.de

**Keywords:** gingival displacement, gingival retraction, artificial gingivitis, digitizing, three-dimensional analysis

## Abstract

This randomized clinical study examined the influence of the gingival condition—healthy versus mild inflammation—on sulcus representation and possible gingival recession for two gingival displacement procedures prior to conventional impression making. The interventions double cord technique or a kaolin paste containing aluminum chloride were applied to 40 probands. The opposite quadrant served as intrapersonal reference (split-mouth design). Precision impressions were then made. Extraoral digitization of the plaster models resulting from the reference impression prior to gingival displacement, the intervention impression and control impressions were the basis for the computer-aided three-dimensional analysis. After six months, a mild artificial gingivitis was induced, and the contralateral quadrant (cross-over design) was examined for the intervention. The gingivitis deteriorated the sulcus representation for the double cord technique group but did not affect the paste technique group. The gingival condition had no influence on the marginal gingiva height changes. The minor extent of those changes, which were measured up to six months after intervention at the palatal study site, were not considered to be in the clinically relevant range for gingival recession. For healthy gingiva, the cord technique showed superior sulcus representation compared to the paste technique. This advantage was lost to a great extent under the conditions of mild gingivitis.

## 1. Introduction

Whenever restorations are necessary for teeth with subgingival margins, sufficient gingival displacement is an essential requirement prior to precision impression making. For this purpose, the impression material must flow into the sulcus in sufficient quantity and to a sufficient depth to enable a precise reproduction of the die [1]. There are various methods for gingival displacement to achieve those aims adequately. Two widely used procedures are the retraction cord technique [2,3,4] and the cordless technique with paste, foam or gel [5]. As an astringent for the retraction cord technique, aluminum chloride and ferric sulfate are very commonly used due to the satisfactory results in terms of draining and bleeding management without systemic side effects as is possible with epinephrines [6]. For the cordless technique, paste, foam or gel are injected into the sulcus for gingival displacement and drainage or hemostasis [7]. A well-known representative is Expasyl (Pierre Roland, France). This is a kaolin clay-based paste with 15% aluminum chloride [5].

A comparison of the retraction cord technique to the cordless aluminum chloride paste technique showed that the cords distended the sulcus further than the paste [2,8,9,10,11]. However, expansion reached with the paste technique was described as still being a clinically sufficient method for gingival displacement. The advantage of the cordless paste technique over the retraction cord technique is greater sparing of periodontal tissue [9,12]. Persisting gingival recession due to prosthodontic treatment should be excluded or at least limited to a minimum. The paste was more effective in achieving this goal [3,9,13,14,15]. In addition, the cordless paste technique was shown to be more comfortable for patients [9,16].

A healthy periodontal or gingival status was a prerequisite in comparative clinical studies between retraction cord and cordless techniques [2,3,8,9,10,11,12,16,17,18,19,20,21,22,23]. Such inclusion criteria eliminate the potential confounding factor of periodontal or gingival inflammation, as inflamed tissue has altered properties and thus may behave differently regarding gingival displacement. In clinical reality, however, it is not uncommon to find a plaque index [24] above zero [25] and mild gingivitis to be present at the time of gingival displacement and impression making. This is the case for patients performing suboptimal oral hygiene or those for whom oral hygiene instructions prior to the treatment with fixed restorations did not show full success. In addition, if the impression is made in the session following the preparation, a provisional acrylic crown, which favors plaque accumulation [26], may lead to gingivitis by the time the impression is made. To the authors’ best knowledge, no information is available to date on how the gingival condition affects the achievable sulcus representation and possible gingival recession when using the two gingival displacement methods, cord displacement technique with astringency and cordless technique with kaolin clay-based paste.

Therefore, the aim of the present clinical study was to determine the influence of the gingival condition—i.e., healthy versus mild inflammation—on the sulcus representation and possible gingival recession for these two gingival displacement techniques.

## 2. Materials and Methods

This randomized clinical trial was approved by the Ethics Committee of Ulm University under the number 113/09 on 17 June 2009. It has been registered in a public trials registry (German Clinical Trials Register, DRKS00003374).

### 2.1. Trial Design

**Figure 1:**
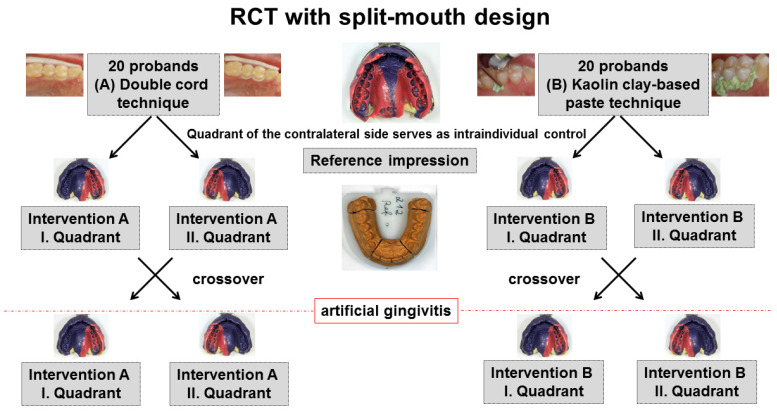
Study design of the randomized clinical trial (RCT) with split-mouth design with the two interventions (**A**) double cord technique or (**B**) kaolin clay-based paste for gingival displacement prior to conventional impression making.

In a randomized, controlled, clinical trial (RCT) with split-mouth design (Figure 1), the interventions (A) double cord technique in combination with the astringent aluminum chloride or (B) a kaolin clay-based paste containing aluminum chloride were randomly allocated and applied to 40 gingivally healthy probands, 20 probands for each intervention. The contralateral quadrant served as intrapersonal reference without gingival displacement prior to impression making (split-mouth design). After six months, artificial gingivitis was induced in each proband and the same intervention was repeated in the contralateral quadrant (cross-over design). A reference impression was made prior to both interventions for each proband. Directly after gingival displacement, an intervention impression was made. Control impressions were made three and six months after each intervention. The study was stratified by gender (m/f). After six probands (three for each intervention), the study design was reevaluated for flaws and it was determined that no adjustments were required. Thus, the study data of these six probands were processed in the same way as the data of the following 34 probands.

### 2.2. Participants

The study was carried out in accordance with the Code of Ethics of the World Medical Association (Declaration of Helsinki) at the Department of Prosthetic Dentistry, Center of Dentistry at Ulm University in Germany. Informed consent was obtained for all probands. The privacy rights of the probands were respected.

The inclusion criteria were the following:

- Between 18 and 80 years of age
- Healthy, not suffering from chronic or acute infections or diseases
- No alcohol or drug abuse
- No known allergies to the materials used (astringent, anesthetics, impression material)
- Presence of both premolars in I. and II. quadrants in the upper jaw with their adjacent teeth
- Premolars and adjacent teeth in upper jaw naturally healthy or restored in a defect-free state with restoration margins located at least 1 mm supragingivally
- Legally competent

The exclusion criteria were the following:

- Pregnancy
- Smoker
- Upper premolar restored with full-coverage crown
- Periodontal Screening Index (PSI; definition by the German Society for Periodontology) above 2 in at least one sextant

### 2.3. Interventions

**Figure 2:**
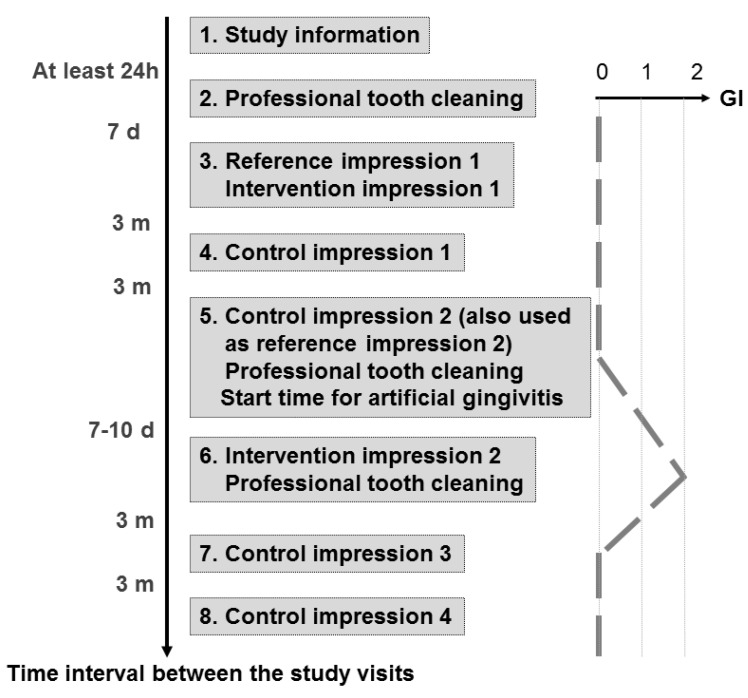
Study procedure in eight study visits with intended course of the gingival index (GI); h: hours; d: days; m: months.

The duration of the study was 12.5 months for each proband. The study was divided into eight study visits (Figure 2), which were performed by two dentists. The volunteers were informed about the objectives of the study and the procedure within the study (Visit 1). The volunteers were checked for the inclusion and exclusion criteria as described above and a detailed finding of both jaws was documented. Volunteers who met the inclusion criteria and gave their written informed consent to participate in the study at least 24 h after the study information were included in the study. The periodontal findings were recorded in the following session (Visit 2) by means of a six point pocket measurement (PD), the Gingival Index (GI [24]) and the Periodontal Screening Index (PSI; definition by the German Society for Periodontology). The PD and GI were also recorded at each subsequent visit. Each proband received professional tooth cleaning, individual oral hygiene instructions and fluoridation.

Seven days after the professional tooth cleaning (Visit 3), any adhering plaque was removed carefully supragingivally with a soft rubber cup without provoking bleeding. First, a reference impression of the upper jaw was made with a customized tray (Optosil Plus, Heraeus Kulzer, Germany). For this purpose, a double mix impression was made with a polyether material (Impregum Penta H DuoSoft Quick (Heavy Body) and Impregum Garant L DuoSoft Quick (Light Body), 3M Oral Care, Germany), whereby both quadrants were syringed with light body material. This impression was allowed to be repeated in case of obvious impression errors. Depending on the randomization, the double cord technique (intervention A) or kaolin paste (intervention B) was applied to both unprepared maxillary premolars of the first or second quadrant as described below.

#### 2.3.1. Intervention A

The primary cord (Retracto, impregnated, Roeko, Germany), a size 1 cord already impregnated with aluminum chloride, was placed proximally and palatally to the premolars. The impregnated cords were chosen in order to ensure an even concentration of the astringent. An unimpregnated cord (Retracto, not impregnated, Roeko, Germany) of size 2 was loosely placed on top of the primary cord. Following clinical routine, the cords remained in place for ten minutes. Before the intervention impression was made, the secondary cords were slightly moistened to avoid tearing off any adhering epithelium and removed. Both primary and secondary cords were always removed before the impression was made. The light body polyether was already injected into the sulcus during cord removal. No intervention was performed in the opposite quadrant, which served as intra-individual reference (cross-over). The premolars of these quadrants were not syringed with light body material.

#### 2.3.2. Intervention B

The sulcus of the premolars was filled proximally and palatally with Expasyl (Pierre Roland, France), a kaolin aluminum chloride paste. The material was injected and was left for five minutes in the sulcus. The material was removed before impression making by thoroughly rinsing with water and suctioning. The premolars of the non-intervention quadrant were not syringed with light body material.

The intervention impressions were not allowed to be repeated to avoid an influence on the gingival retraction. Faulty interventional impressions would have led to censoring of the case, which did not happen in the study. After impression disinfection and elastic recovery of the impression of at least four hours, saw models were made with class IV synthetic super hard stone according to the Giroform process (Amann Girrbach, Germany). The area of the two premolars and their adjacent teeth was segmented to minimize plaster expansion. While both quadrants of the reference model were saw-cut, only the quadrant of the intervention side of the intervention model was sawn. All models of the study were blinded prior to the analyses.

The first follow-up visit after the first intervention was performed three months later (Visit 4). Any adherent plaque was removed supragingivally with a soft rubber cup. A control impression was made (one-stage impression technique) and a control plaster model was fabricated.

The second follow-up after the first intervention was performed another three months later (Visit 5). The same examinations were performed, and control impression No. 2 was made, which was also used as reference impression No. 2. A professional tooth cleaning was performed after impression making. Subsequently, artificial gingivitis was induced by instructing the proband not to perform dental hygiene until the next visit. In detail, the proband was instructed not to use mouth rinses or brush teeth. In this way, it should be achieved that the GI, which had been ideally at a value of zero up to the present visit, rose to a value of two by the next visit, corresponding to a mild manifestation of gingivitis.

After the induction of the reversible experimental gingivitis 7–10 days later (Visit 6), the second intervention was performed with the same gingival displacement technique in the contralateral quadrant (cross-over design). The other maxillary quadrant again served as a control. After impression making, the plaster model was fabricated in the same manner as described above and, subsequently, the proband received a professional tooth cleaning, aiming for an again healthy gingiva, represented by a decreased GI value of zero by the next visit.

The first follow-up after the second intervention was performed after three months (Visit 7). The procedure was the same as for Visit 4.

The second follow-up after the second intervention (Visit 8) was again three months later and was also the final examination. The procedure was the same as for Visit 4 and Visit 7.

### 2.4. Outcomes

The primary outcome was the sulcus representation (difference measurements between digital data of the intervention models and the reference models in mm). The secondary outcome was the marginal gingiva height change (in mm), determined by comparison between the digital data of the control models and the corresponding reference model.

All models (reference, intervention and control models) were digitized using a non-contact optical method with a measurement uncertainty as given by the manufacturer of ~16 µm (DigiSCAN L, AmannGirrbach, Germany). The reference models were first digitized without exposing the subgingival impression border. Then the impression border of the reference models was exposed under a light microscope with 25-fold magnification (Leica MS5 dental stereo microscope with EC3 digital camera)—the same was done for the intervention models—since the data sets of these models were used to analyze the sulcus representation. For this purpose, a diamond bur with a diameter of 0.8 mm was used after the rough exposure. The impression border of the control models was not exposed, since the data sets of these models and of the reference models prior to exposure were used to record the marginal gingiva height change. The only exception was the second control model, which served both as control model 2 (before exposure) and as reference model No. 2 and was thus subsequently exposed. Before digitizing, all model errors (plaster beads, etc.) were blackened under the microscope. These blackened areas are not captured by the scanner. A digitizing system-specific filtering of the acquired data sets took place, i.e., outliers and scatter points were removed to increase data quality (Argus, Fraunhofer Institut IOF, Germany). Subsequently, reverse engineering was carried out (ce.novation, ILMCAD, Germany) to obtain a spatial orientation (top side, bottom side, surface normals) instead of disordered points. The correct alignment of the data sets to each other in a common analysis coordinate system (best-fit registration) by computer-aided design (CAD) software (Geomagic Studio 9 and Qualify 9, Geomagic, Research Triangle Parc, NC, USA) was crucial for the subsequent three-dimensional (3D) analyses. The quality of the assignment was determined by the RMS error (root mean square error) and should be less than 32 µm, corresponding to the measurement uncertainty of the digitizing system for complete arch models.

#### 2.4.1. Sulcus Representation

**Figure 3:**
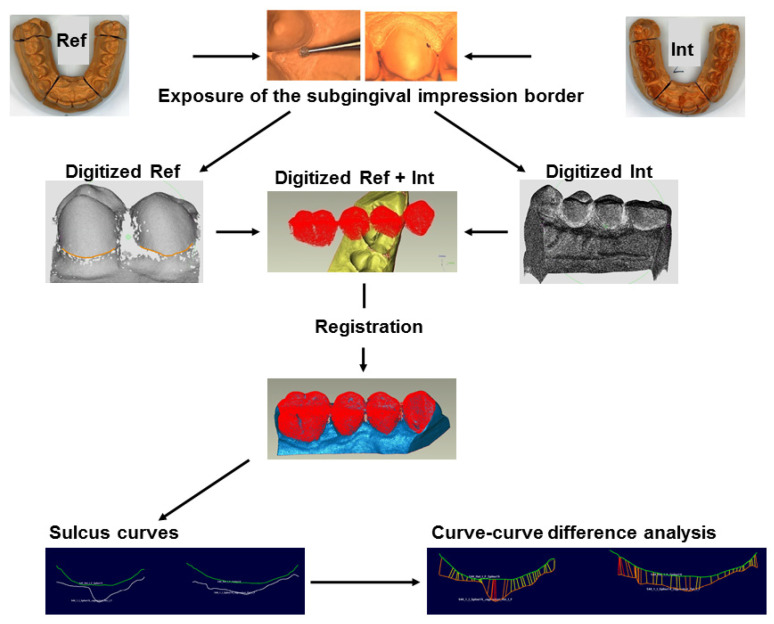
Sulcus representation analysis; Ref: Reference model; Int: Intervention model.

For the analyses of the sulcus representation (Figure 3), the data set of the quadrant of interest (intervention or control quadrant) of the intervention model was aligned to the data set of the corresponding, exposed reference model. Boundary curves along the impression border, i.e., the bottom of the exposed sulcus were constructed (ce.novation, ILMCAD, Germany) at all four upper premolars along the exposed impression border of the reference as well as the intervention model. The difference in sulcus representation between the two boundary curves of the superimposed models (reference versus intervention model) was calculated with CAD software (Surfacer 10.6, Imageware Inc., Ann Arbor, MI, USA) for each tooth (curve-curve-difference). By definition, a more apical sulcus representation of the intervention impression compared to the reference impression, i.e., capturing more of the sulcus, resulted in a negative value. Thus, negative values indicated a better sulcus representation for the intervention impression, positive values indicated a better sulcus representation for the reference impression.

#### 2.4.2. Marginal Gingiva Height

**Figure 4:**
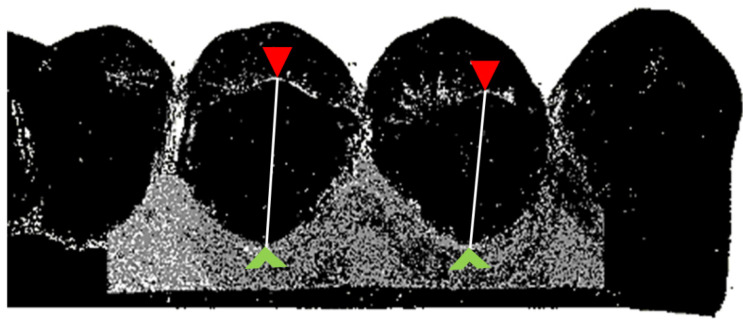
Marginal gingiva height change analysis. Schematic view of distance measurement for the marginal gingiva height change analysis (palatal aspect of upper premolars). Red arrowhead points to the highest point of the palatal cusp tip, green arrowhead points to the deepest point of the palatal marginal gingiva.

For the analyses of the marginal gingiva height change (Figure 4), the data sets of the four unexposed control models were assigned to the data set of the respective last unexposed reference model in terms of time. Thus, the marginal gingiva height change was analyzed for time spans up to six months after intervention. Distance measurements were used to measure the marginal gingiva height change (Geomagic Studio^®^ 9 and Qualify^®^ 9, Geomagic Inc, Research Triangle Park, NC, USA). The occlusal–crestal measurement direction corresponded to the Z-axis.

A hard tissue measurement point was set at the highest point of the palatal cusp tip for each of the four premolars of the reference model. These hard tissue measurement points were transferred from the reference model to the aligned control models. A soft tissue measurement point was set at the deepest point of the palatal marginal gingiva for each of the four premolars (reference and control models). Then the distances between hard and soft tissue measurement points was gauged. The marginal gingiva height change was determined by subtracting the measured distances of the control models, three and six months after intervention, from those of the corresponding reference model. A positive value corresponded to a gain of marginal gingiva height; a negative value corresponded to a loss of marginal gingiva height.

### 2.5. Sample Size

As no information was available at the time of the study design for the kaolin clay-based paste material regarding the expected sulcus exposure and marginal gingiva height change, no formal sample size calculation could be performed. Therefore, this was an explorative pilot study for which the number of cases was set at 40 subjects based on the experience in similar studies.

### 2.6. Randomisation

Stratified block randomization was performed. The 40 subjects were randomized according to the intervention (gingival displacement technique) and the quadrant of the first intervention without gingivitis. This resulted in four randomization groups (*n* = 10) with intervention A (cord) or B (paste) applied for the first time in the first or second quadrant. The study was stratified into the two gender groups male/female. Randomization concealment was ensured by assigning a non-participant of the study to perform the allocation according to the randomization list, which consisted of a purely randomized sequence (computer-generated random numbers). The person responsible for the randomization assigned the proband according to the randomization list and filled in the information on the quadrant of the first intervention and the gingival displacement technique to be used on the case report file (CRF). Before the end of the recruitment phase, in the event of dropouts, a new volunteer was recruited.

### 2.7. Blinding

Due to the clearly distinguishable measures (cords versus paste), blinding of dentist and proband was not possible. However, the evaluation was consistently blinded. For this purpose, all models (reference, intervention and control models) were blinded before digitizing and the subsequent analyses. Thus, the respective co-authors performing these study parts did not know which intervention group was involved. Also, the biometrician performed the statistical analysis with the still-blinded data.

### 2.8. Statistical Methods

In case of continuous outcomes, both mean together with standard deviation and boxplots were used for description. Categorical data were analyzed using absolute and relative frequencies. Differences in the primary and secondary outcomes were investigated via the two-sided one-sample *t*-test and the calculation of mean differences and the respective two-sided 95% confidence interval. To account for statistical dependencies between different teeth and quadrants within the same subject, linear mixed effects regression models were applied for the primary outcome. The regression models were calculated separately for both intervention materials (cord/paste). In each regression model, the explanatory variables were as follows:

- Point in time (including the gingival condition (healthy/mildly inflamed))
- Tooth (first/second premolar)
- Quadrant (first/second quadrant)

For each of the following influencing variables separate mixed effects regression models were investigated:

- Intervention (yes/no)
- Gender (female/male)
- GI-value
- PSI-value
- Maximum pocket depth (out of six-point pocket measurements)

Afterwards, the regression analyses (primary outcome) were calculated again, excluding all cases in which the gingival index (GI) deviated from the targeted values. Cases were excluded if the GI was above 1 at the first intervention (expected value: 0) and if the GI was below 2 at the second intervention after generation of the artificial gingivitis (expected value: 2).

All results from the statistical tests were considered as significant, if *p* < 0.05. The statistical analysis was carried out using SAS version 9.4 (SAS Institute, Cary, NC, USA) and IBM SPSS Statistics 27 (SPSS Inc., Chicago, IL, USA). Because of the explorative nature of this study, all results from statistical tests have to be interpreted as hypothesis generating only and not as confirmatory.

## 3. Results

### 3.1. Participant Flow

**Figure 5:**
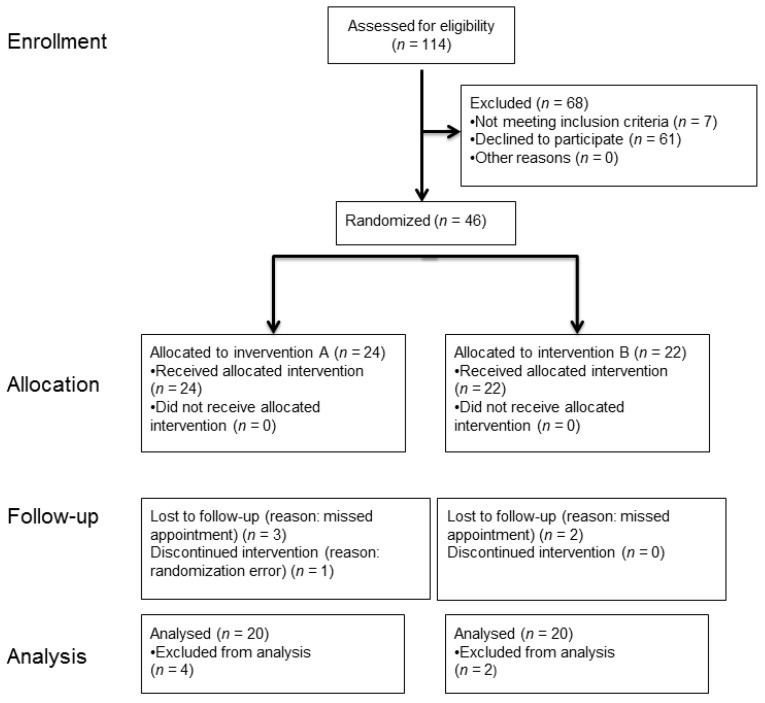
Study Flow Diagram according to CONSORT 2010.

A total of 114 individuals were assessed for eligibility, 46 were randomized (Figure 5). During the recruitment phase, dropout from the study occurred six times after randomization. Five cases were assigned to the reason “missed appointment”. For one proband, a randomization error occurred: a female volunteer was mistakenly entered in a column for male volunteers in the randomization list.

No further dropout occurred after recruitment was completed. In both groups, all volunteers received the intended treatment.

### 3.2. Recruitment

The recruitment phase began in June 2009 and ended in October 2010, after the intended number of subjects (*n* = 40) had been recruited. The last control impression was made in November 2011 (last patient out).

### 3.3. Baseline Data

The demographic and clinical characteristics were compared between the intervention groups (Table 1) and did not differ significantly between the two groups. At the time of the first intervention, the desired GI of maximum 1 was present in 18 probands of group A (cord) and 19 probands of group B (paste). At the time of the second intervention after 7 to 10 days of oral hygiene abstinence, the desired GI of 2 was present in 18 probands of group A and 16 probands of group B. Due to stratification by gender, the gender distribution was even in both groups (50% female, 50% male). The range of age was between 20 and 45 years for the cord group and between 18 and 54 years for the paste group.

### 3.4. Numbers Analyzed

For primary and secondary outcome, all 40 probands were included in the analyses, 20 for each gingival displacement technique.

For the sulcus representation, all data sets were available. For the marginal gingiva height change, four values were missing:

- Two values (both premolars) for the control quadrant for B (paste group) at the 3-month follow-up visit after the second intervention.
- Two values (both premolars) for the intervention quadrant for A (cord group) at the 6-month follow-up visit after the second intervention.

For the statistical analyses excluding all cases with deviating GI values, data of nine probands were no longer included:

- GI value of 2 at the first intervention (A: two probands; B: one proband)
- GI of 1 at the second intervention (A: two probands; B: four probands)

### 3.5. Outcomes

#### 3.5.1. Sulcus Representation (Primary Outcome), *t*-Test for Dependent Samples

##### Influence of an Intervention Performed

**Figure 6:**
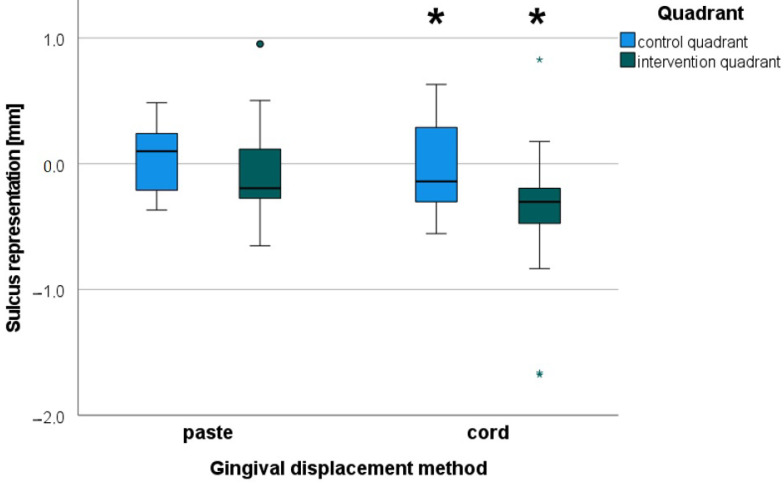
Sulcus representation for control quadrant (before inducing gingivitis at Visit 5) versus intervention quadrant (first intervention); negative value: displacement of the sulcus apically compared to reference impression; * (large asterisks): significant (*p* < 0.05); the circle represents an outlier value (more than 1.5 times the box width away); the little asterisks represent extreme values (more than 3 times the box width away).

The split-mouth design allowed a comparison of the sulcus representation between the intervention quadrant and the control quadrant (Figure 6). As expected, there was more gingival displacement in the intervention quadrant than in the control quadrant. The *t*-test showed that the improvement in sulcus representation by the “cord” intervention over no intervention was significant (*p* < 0.01), whereas this was just not the case for the “paste” intervention (*p* = 0.06).

##### Influence of the Gingival Displacement Method

**Figure 7:**
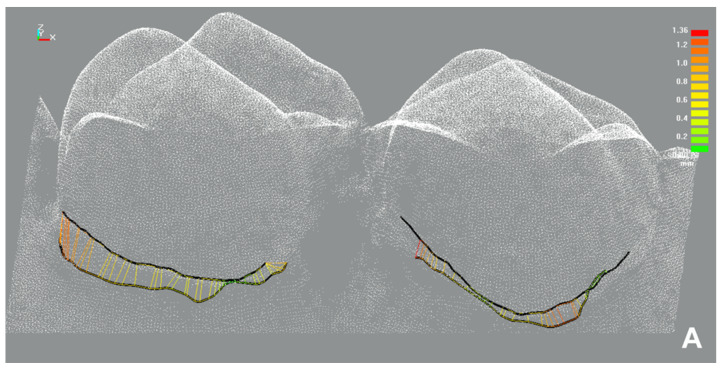

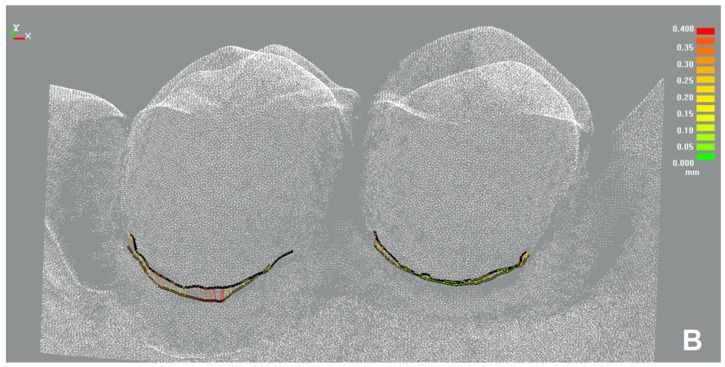
Curve–curve difference analysis as part of the sulcus representation analysis; view on digitized upper premolars (palatal aspect); color-coded difference images for the cord technique (**A**) and the paste technique (**B**).

The mean values with 95% confidence intervals of the achieved sulcus representation are summarized in Table 2. The cord technique showed better sulcus representation for the healthy gingiva and only slightly better sulcus representation for the artificial gingivitis than the paste technique (Table 2). Figure 7 gives examples for typical color-coded difference images of the curve-curve-difference analyses for both gingival displacement methods.

##### Influence of Gingival Condition

The results for the sulcus representation were compared between the two gingival conditions (Table 3). The paste technique was hardly affected. The cord technique was affected more showing worse sulcus representation in case of gingivitis.

#### 3.5.2. Sulcus Representation (Primary Outcome)-Regression Analysis

The results for the sulcus representation after application of cord or paste using linear mixed effects regression analyses showed no significant influence of any influencing variable for the paste technique. For the cord technique, the gingival condition (effect: point in time) showed a significant influence when considering the influencing variables intervention, gender, GI value, PSI value and maximum pocket depth (*p* < 0.01). No significant influence could be determined for tooth (first or second premolar) and quadrant (first or second quadrant) for either displacement method (cord/paste).

##### Regression Analysis Excluding Cases with Strongly Deviated GI

After excluding the above mentioned nine subjects due to a deviating GI value, the regression analysis was repeated. The results remained unchanged for both groups.

#### 3.5.3. Marginal Gingiva Height Change (Secondary Outcome)-*t*-Test for Dependent Samples

**Figure 8:**
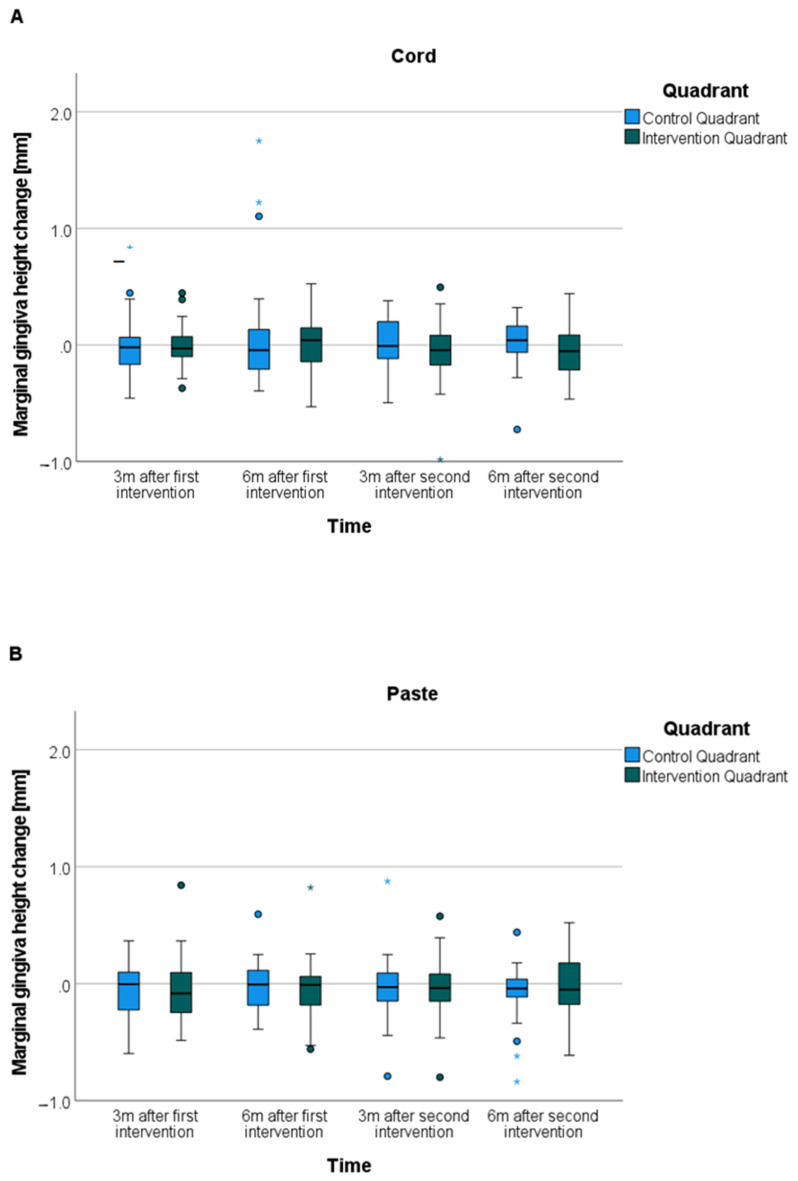
Marginal gingiva height change for control quadrant (no intervention) versus intervention quadrant ((**A**) cord; (**B**) paste) for three months (3m) and six months (6m) after first intervention (healthy gingiva) and second intervention (artificial gingivitis); the circles represent outlier values (more than 1.5 times the box width away); the asterisks represent extreme values (more than 3 times the box width away).

The split-mouth design allowed a comparison of the results between the intervention quadrant and the control quadrant (Figure 8). The marginal gingiva height did not change significantly (*p* = 0.23) between the control quadrant and the intervention quadrant for either gingival displacement method, neither three months, nor six months after the first or second intervention for the first and the second premolar. Both gingival displacement techniques showed mean values of marginal gingiva height change less than or equal to 0.05 mm.

### 3.6. Ancillary Analyses

The RMS (root mean square) error was analyzed as a measure of the quality of alignment (best-fit registration). The analysis proved a high quality of alignment (Table 4).

### 3.7. Harms

No severe adverse events (SAEs) occurred in the clinical study. The measured marginal gingiva height changes were palatal and of such low extent, that they could be considered clinically unproblematic.

## 4. Discussion

The aim of the present clinical study was to determine the influence of the gingival condition—i.e., healthy versus mild inflammation—on sulcus representation and possible occurrence of gingival recession for two gingival displacement techniques: the cord displacement technique with astringent and the cordless technique with a kaolin clay-based paste. The mildly inflamed gingiva deteriorated the sulcus representation after applying cords for gingival displacement. It did not affect the lower sulcus representation after applying the paste technique. The gingival condition had no influence on the marginal gingiva height change for either gingival displacement method.

The cord technique showed a superior gingival displacement compared to the aluminum chloride paste technique for a healthy periodontal and gingival status (first intervention). This finding is in accordance with the findings of other studies [2,8,9,10,11,27], including only patients with a healthy periodontal or gingival status. However, the definition of the gingival displacement differed. In the study at hand, it was quantified in the vertical direction (sulcus representation), i.e., the displacement of the sulcus to apical was investigated. In the vast majority of studies, the horizontal retraction was measured. The vertical displacement has rarely been quantified in studies comparing the cord and paste technique. Gupta et al. [27] also quantified the vertical displacement, but used a different measuring method as described further below. They also found that the cord technique showed superior vertical displacement compared to the paste Expasyl. For the sake of completeness, another definition for vertical tissue displacement is to be mentioned. The displacement of the gingival crest—not the sulcus—in the apical direction can also be considered as vertical tissue displacement [23,28,29]. This outcome is not comparable to that of our study. Although the vertical displacement of the sulcus to apical has hardly been studied, it is crucial to expose the uncut portion of the tooth apical to the finish line in case of prepared teeth [1]. Therefore, our study also adds valuable information to the field of research on cord versus paste technique performance for healthy gingival conditions: cords support the impression material to flow deeper into the sulcus and thus are potentially more reliably below a subgingival finish line. For the second intervention with mild gingivitis, this advantage of the cord technique was lost to a great extent, and it was hardly more effective than the paste technique. Further studies on the influence of gingivitis, including additionally the quantification of the horizontal displacement, seem reasonable. If it is confirmed that the cord technique generally loses most of its advantage of a better gingival displacement in cases of mild gingivitis, then the aluminum chloride paste technique can also be selected more frequently in mild gingivitis. This can be done in order to use its known advantages, such as better hemorrhage control [13,27,30], especially against the background of gingivitis.

The minor extent of the marginal gingiva height changes for both gingival displacement groups and for both gingival conditions cannot be considered as a clinically relevant gingival recession. The values after intervention were comparable to those without any intervention, for which gingival recession can be excluded. These results were obtained mid-palatally at the upper premolars up to six months after gentle application of the double cord or paste technique. They are in contrast with the findings of other comparative studies, which showed that the paste technique was more effective in preventing recession than the cord technique [3,9,13,14,15], which led to—mostly mild—recessions. The reasons for different results may lie in different study designs. Whereas we measured the marginal gingiva height change after three and six months, most comparative studies quantified this outcome for the cord and paste group up to a maximum of one month after gingival displacement [14,15] or prosthesis delivery [3]. Another study measured the gingival recession at one and three months after gingival displacement [13]. The timing of measurement influences the results. The values decreased from 14 to 28 days [14] and rose from the one-month to the three-months follow-up examinations for both techniques in another study [13]. The location of the measurement also differed between the studies: other researchers quantified the buccal recession [13,14,15]. The buccal and palatal gingiva may act differently after the application of gingival displacement procedures. In our study, neither gingival displacement method led to a gingival recession for healthy gingiva, which was also a prerequisite in the comparative studies mentioned above, but also for mildly inflamed gingiva.

We applied the typical procedure for the induction of an artificial gingivitis: first a pre-induction period with professional tooth cleaning, then an induction period with waiving of oral hygiene and finally a resolution period to terminate the gingivitis by restarting oral hygiene. Our induction period of 7–10 days is in the lower range of the typical induction periods for artificial gingivitis according to a meta-analysis of clinical studies with induction periods ranging from 10 days (4 out of 22 included studies) to 28 days, with 21 days being the most common period [31]. After seven days, changes of gingival indices and biochemical markers compared to the baseline data could already be detected [32,33,34,35,36]. Accordingly, in the present work, we achieved an increase in the gingival index. For some probands, however, the intended increase could not be reached. This could be explained either by a lack of compliance, i.e., the probands did not adhere to the instructions. Another plausible explanation is that the probands were so-called “slow responders” who responded with a delay in the induction phase [32,34]. However, this was biometrically adjusted by repeating the analyses excluding all cases in which the gingival index deviated strongly from the target course. This did not result in any changes in the results. The short induction period was deliberately chosen. The aim of the present study was to produce only mild gingivitis in which an impression is still reasonable. This scenario occurs in the clinical reality, if the impression is not made until the following session. A provisional acrylic crown, which favors plaque accumulation [26], may lead to mild gingivitis after a short wearing period of about one week. Even if the impression is made at the preparation appointment, many patients have a gingival index greater than 0 due to less-than-optimal oral hygiene. This was also evident in the present study. Although most of the probands were dental students, who are assumed to have a high level of awareness regarding oral hygiene, 25 volunteers had a GI of 2 at the time of recruitment, 14 volunteers had a GI of 1 and only one volunteer had a GI of 0. The GI decreased with professional tooth cleaning in our study. However, professional tooth cleaning is not always performed before prosthetic treatment in the clinical reality. These baseline data of the GI underline the relevance of the present study to investigate the performance of the two gingival displacement methods for mild gingival inflammation.

Both cords were removed before making the impression. The actual effect of the gingival displacement techniques was to be measured in the vertical direction. Removal of the cords ensured complete visualization of the entire sulcus. Furthermore, the decision to remove both cords was due to the comparability between the two techniques. The paste Expasyl is also completely washed out of the sulcus before impression making. In case of the double cord technique, however, the primary cord is often left in the sulcus during impression making in the clinical reality. This allows persistent expansion of the sulcus during impression making. However, this factor is hardly an influencing factor in the study, since the light body polyether was already injected into the sulcus during cord removal. Earlier studies on this subject showed that after the use of the double cord technique, the sulcus still exhibits an expansion of approximately 0.2 mm in the proximal sulcus area after 20 s [37,38]. We were below the value of 20 s according to the immediate injection of the impression material.

We used a computer-aided three-dimensional analysis for quantification. Gupta et al. [27] also quantified the vertical displacement for the cord versus the paste technique. Therefore, they used flexible measuring strips with 0.5 mm grading to measure the sulcus depth clinically before and after the application of the gingival displacement techniques at one specific site of the study tooth. The authors pointed out a certain inaccuracy due to that method. For our method, the accuracy of the concatenation of impression making, model fabrication, digitization and assignment of the data sets, as well as exposure of the impression border and setting of the measurement points is relevant. Due to the high degree of standardization, the resulting errors are minimized and are in the range of ± 40 to 50 micrometers [39,40]. The quality of assignment of the data sets was quantified specifically for this study (root mean square error) and proved to be high. Another advantage next to the high accuracy is the objectivity of the procedure. The curve–curve analysis was done automatically by the software. Furthermore, it was done over the entire palatal half. As the sulcus representation varied notably along this half, a measurement at only one single site may produce inconclusive results.

## 5. Conclusions

The following conclusions can be drawn for the cord technique in combination with the astringent aluminum chloride and the kaolin paste Expasyl for gingival displacement prior to conventional impression making:

- For healthy gingiva, the cord technique achieved a better gingival displacement in vertical direction (sulcus representation) than the paste technique, with the impression material flowing deeper into the sulcus for the cord technique.
- Mild gingivitis worsened the sulcus representation when using the cord technique but had no influence on the sulcus representation of the paste technique. This means that the advantage of the cord technique with regard to a better sulcus representation was lost to a great extent under the condition of mild gingivitis.
- Neither cord nor paste technique showed—with gentle application in this study—permanent gingival recessions at the palatal study sites up to six months, neither for healthy nor for mildly inflamed gingiva.

## Figures and Tables

**Table 1:** Demographic and clinical characteristics for group A (cord) and group B (paste); * one-sample *t*-Test for dependent samples (Variance homogeneity (Levene) given); PSI: Periodontal Screening Index; GI: Gingival Index; SD: Standard Deviation; n.s.: not significant (*p ≥ 0.05*).

	Group A (Cord; *n* = 20) (Mean ± SD)	Group B (Paste; *n* = 20) (Mean ± SD)	*p*-Value *
Age (baseline)	25.85 ± 6.82	27.40 ± 9.91	0.57 (n.s.)
PSI (baseline)	1.65 ± 0.49	1.55 ± 0.61	0.57 (n.s.)
GI (first intervention, healthy gingiva)	0.75 ± 0.64	0.55 ± 0.61	0.32 (n.s.)
GI (second intervention; artificial gingivitis)	1.90 ± 0.31	1.80 ± 0.41	0.39 (n.s.)

**Table 2:** Mean difference values in millimeters with lower (LL) and upper limit (UL) of 95% confidence interval (CI) of the sulcus representation for both gingival displacement methods, separately for healthy gingiva (HG) and artificial gingivitis (AG).

	Healthy Gingiva			Artificial Gingivitis		
Gingival Displacement Method (HG: *n* = 40; AG: *n* = 40)	Mean Difference [mm]	LL 95%CI	UL 95%CI	Mean Difference [mm]	LL 95%CI	UL 95%CI
Cord	−0.37	−0.50	−0.24	−0.18	−0.26	−0.10
Paste	−0.10	−0.20	−0.00	−0.07	−0.15	0.01

**Table 3:** Influence of the gingival condition (healthy gingiva (HG) versus artificial gingivitis (AG)) on the sulcus representation for the cord and the paste technique; *: *t*-Test for Dependent Samples; n.s.: not significant (*p ≥ 0.05*).

	Group A (Cord; *n* = 20) HG versus AG		Group B (Paste; *n* = 20) HG versus AG	
	First premolar	Second premolar	First Premolar	Second Premolar
*p*-value *	0.02	0.07 (n.s.)	0.37 (n.s.)	0.74 (n.s.)

**Table 4:** Root mean square (RMS) error for the sulcus representation (analysis of all second intervention cases) and for the marginal gingiva height change (analysis of all first intervention cases). SD: Standard Deviation.

	Sulcus Representation	Marginal Gingiva Height Change
Mean RMS Error ± SD	14.6 µm ± 4.5	20.5 µm ± 8.2
Percentage of RMS Errors Below the Threshold of 32 µm	100%	96.3%

## Data Availability

The data presented in this study are available on request from the corresponding author.
